# Stool Dynamics and the Developing Gut Microbiome During Infancy

**DOI:** 10.1177/07487304251407313

**Published:** 2026-01-20

**Authors:** Mohammed Al-Andoli, Sarah Schoch, Andjela Markovic, Christophe Mühlematter, Matthieu Beaugrand, Oskar G. Jenni, Rabia Liamlahi, Jean-Claude Walser, Dennis Nielsen, Salome Kurth

**Affiliations:** * Department of Psychology, University of Fribourg, Fribourg, Switzerland; † Department of Pulmonology, University Hospital Zurich, Zurich, Switzerland; ‡ Center of Competence Sleep & Health Zurich, University of Zurich, Zurich, Switzerland; § Child Development Center, University Children’s Hospital Zurich, Zurich, Switzerland; || Genetic Diversity Center, ETH Zurich, Zurich, Switzerland; ¶ Department of Food Science, University of Copenhagen, Copenhagen, Denmark

**Keywords:** precision medicine, intestinal microbiota, circadian rhythm, fecal dynamics, beta diversity, relative abundance, child maturation

## Abstract

The infant gut microbiome is a dynamic ecosystem, and it is key to early development, immune maturation, and overall health. Recent insights reveal that the gut microbiota undergoes changes across the 24-h day, raising the possibility that it may act as a “zeitgeber,” supporting the host’s sleep-wake organization. Despite its importance, timing factors influencing microbiome composition are poorly understood, limiting its use as a health indicator. This study investigates the relationship between stool dynamics (defecation interval, time of sampling), sleep pressure (interval since last sleep), meal timing, and gut microbial composition. Stool samples from 198 healthy infants, aged 3 to 31 months, were analyzed to assess microbial diversity, richness evenness, and abundance. Our findings reveal that longer intervals between bowel movements are associated with increased microbial diversity, evenness, and richness. Stool timing is associated with shifts in microbial composition, especially in younger infants, indicating diurnal microbial fluctuations to become more stable as infants mature. Longer periods of wakefulness were associated with increased microbial diversity in early infancy, although this effect appeared to diminish with age. Feeding schedules had limited effects on the gut microbiome. Longer fasting before sampling showed no significant associations with most microbial parameters, except for a positive association with microbial richness. At the phylum level, results indicate that infant gut microbial composition is influenced by behavior and physiology. Longer intervals between bowel movements were associated with shifts in bacterial abundance, with *Proteobacteria* decreasing and *Actinobacteria* increasing. In addition, later stool sampling times revealed higher *Actinobacteria* levels, and longer fasting was associated with reduced *Bacteroidetes*. Sleep pressure showed a trend effect with *Firmicutes* displaying a slight decrease in infants who had been awake longer. Our findings underscore the importance of time-based factors on infant gut microbiome composition.

The infant gut microbiome plays a key role in early development, influencing overall health by supporting immune system maturation, metabolic processes, brain development, and sleep regulation (Carlson et al., 2018; Moore and Townsend, 2019; Rodríguez et al., 2015). The gut microbiome shows substantial variation between individuals during the first year of life (Heppner et al., 2023; Matenchuk et al., 2020), influenced by factors such as feeding type, delivery method (e.g., vaginal or cesarean), and antibiotic use (Rutayisire et al., 2016). Diurnal factors, such as diet and sleep pattern, also influence gut microbiome dynamics (Heppner et al., 2023; Matenchuk et al., 2020). Yet despite its critical role in overall health, many determinants of microbiome development still remain poorly understood. Gaining deeper insight into the complex interactions between the gut microbiome, stool dynamics, meal timing, and sleep could lead to more personalized approaches in pediatric and clinical care.

The human gut microbiota is a complex ecosystem composed of bacteria, eukaryotes, archaea, and viruses, including bacteriophages (phages) that target bacteria (Townsend et al., 2021). This microbial community plays essential roles in protecting against pathogens, modulating the immune system, producing vital metabolites, and nourishing gastrointestinal cells (van Best et al., 2015). Crucially, a significant portion of the microbiota undergoes rhythmic oscillations, with up to 60% of its composition fluctuating periodically (Thaiss et al., 2014). In mice, around 20% of commensal species exhibit diurnal variations, and a similar trend is observed in humans, where 10% of species show fluctuations across the day (Thaiss et al., 2014). Specific microbial taxa, such as *Clostridiales*, and *Bacteroidetes* in mice, and *Parabacteroides*, *Lachnospira*, and *Veillonella* in humans, are known to follow these oscillations (Kaczmarek et al., 2017). These findings suggest that the gut microbiota may play a role in the host’s internal clock, with microbiota rhythmicity becoming more pronounced with age and influenced by early-life dietary patterns (Heppner et al., 2024).

Factors such as the timing of sleep, feeding, and bowel movements are closely linked to diurnal or circadian biology, likely influencing both the function and composition of the gut microbiome (de Oliveira Melo et al., 2024; Gombert et al., 2019). Circadian rhythms are internal biological processes that coordinate physiological functions across a 24-h period. While the central pacemaker in the suprachiasmatic nucleus (SCN) regulates systemic timing, peripheral clocks operate in various organs, likely including the gastrointestinal tract, where they influence metabolic and microbial activity (Matenchuk et al., 2020). Internal circadian rhythmicity is calibrated by external “zeitgebers,” contextual cues that support rhythms of physiological processes and thus also the behavioral rhythmization of sleep-wake organization (Mühlematter et al., 2023). In adults, disruptions to circadian rhythmicity have been linked to alterations in gut microbiota composition, which can negatively impact metabolic health (Sletten et al., 2020; Zhao et al., 2022).

The complex relationship between the developing gut microbiome and its surrounding environment—including factors like the specific clock time of stool collection and the frequency of bowel movement patterns—is an area of growing scientific interest (de Oliveira Melo et al., 2024; Matenchuk et al., 2020). Early infancy represents a critical window when the gut microbiota undergoes rapid growth and development (Heppner et al., 2024), and yet despite its importance for several pathways of health, the influence of stool dynamics on microbiome composition remains largely unexplored. These interactions are generally poorly understood at any age of the human lifespan, yet infants, with their frequent bowel movements, offer an excellent model for examining these dynamics. This study aims to quantify the effect of temporal dynamics on infant gut microbiome composition. Specifically, this study investigates how stool dynamics (interval since last defecation, clock time of stool collection), alongside sleep pressure and meal timing, shape the composition of the gut microbiome during the most critical developmental window. We hypothesize that these temporal factors significantly shape microbial composition. Data from 198 healthy infants aged 3 to 31 months were analyzed, and 504 of their stool samples served as basis to characterize these associations.

We hypothesize that early infancy features a highly responsive microbiome that is sensitive to variations in the most fundamental zeitgebers, with sleep pressure and fasting periods being key factors determining individual variability of gut microbiome composition (Mühlematter et al., 2023). Furthermore, as infants mature, we expect the microbiome to stabilize, becoming progressively less susceptible to external deviations and developing stronger internal rhythmicity. Accordingly, we anticipate that the timing factors (bowel frequency, stool timing, sleep pressure, fasting) affect the microbial community structure with age-specific effects, including dynamics in the relative abundance at the phyla level (*Proteobacteria*, *Actinobacteria*, *Firmicutes*, *Bacteroidetes*).

## Methodology

### Participants

This study included 198 healthy infants residing in Switzerland, assessed for sleep-wake patterns and bowel movements at various ages between 3 and 31 months. The predominant assessment ages were 3 months, 6 months, and 12 months (cohorts SDEGU, SPIN, NUTR), while one cohort (SLEEPY) was assessed at varying ages within 5 to 31 months. All infants were generally healthy, born full-term via vaginal delivery and had not received antibiotics during their first 3 months of life (Table 1). In addition, none of the mothers reported a history of gut-related disorders such as irritable bowel syndrome (IBS), inflammatory bowel disease (IBD), or *Clostridioides difficile* infection (Schoch et al., 2022). In addition, more detailed overview of the inclusion and exclusion criteria is available in Schoch et al. (2020). Participants were distributed across the 4 cohorts as follows: SDEGU = 162, SPI*n* = 12, NUTR = 12, and SLEEPY = 14 (Table 2) (Mühlematter et al., 2025). Demographic information, including antibiotic use and feeding type, was available for the SDEGU, SPIN, and NUTR cohorts, representing 92% of all samples, while such data were not available for the SLEEPY cohort.

**Table 1.** Participant demographics at 3, 6, and 12 months, including antibiotic use and feeding type.

(a) Antibiotic Use	Age 3 Months	Age 6 Months	Age 12 Months
- Never	100%	97.2%	92.1%
- Reported intake	0%	2.8%	7.9%
(b) Feeding Type			
- Exclusive breastfeeding	78.2%	42%	22.1%
- Exclusive formula feeding	0%	8.9%	51.7%
- Mixed feeding	21.8%	48.5	15.9%
- Others	0%	0.6	10.3%

**Table 2.** Overview of cohorts, number of infants, and number of stool samples.

Cohort	Number of Infants	Samples Included	Age 3 Months	Age 6 Months	Age 12 Months	Age 5-31 Months
SDEGU	160	389	117	135	137	-
SPIN	12	43	19	24	-	-
NUTR	12	31	16	15	-	-
SLEEPY	14	41	-	-	-	41

The study was conducted in accordance with ethical guidelines, with approval granted by the Cantonal Ethics Committees, Switzerland (BASEC 2016-00730, 2019-02250). All parents or primary guardians provided written informed consent after receiving a comprehensive explanation of the study procedures, ensuring compliance with principles outlined in the Declaration of Helsinki.

### Experimental Design

A total of 504 stool samples were collected from 4 independent cohorts (Table 2): SDEGU (*n* = 389, from 160 infants, 117 at age 3 months, 135 at age 6 months, 137 at age 12 months), SPIN (*n* = 43, from 12 infants, 19 at age 3 months, 24 at age 6 months), NUTR (*n* = 31, from 12 infants, 16 at age 3 months, 15 at age 6 months), and SLEEPY (*n* = 41, from 14 infants, 5-31 months, with a mean sampling age of 12.2 ± 6.2 months, mean ± SD). Parents utilized disposable pipettes and spatulas to collect stool samples from diapers, which were then stored in sterile tubes inside protective bags for temporary refrigeration. Within 72 h, the samples were transported in cooling boxes to the laboratory, where aliquots were stored at −50 °C or −80 °C for subsequent analysis (Schoch et al., 2022). Data on gut microbiota composition were obtained from stool samples through 16s rRNA gene amplicon sequencing, targeting the V3 region to characterize bacterial communities. Contextual information for each stool sample was either provided by the parents or derived from a 24-h diary they completed across several continuous days during the study (Schoch et al., 2022).

Our analysis focused on 4 key factors that could potentially create variance in infant gut microbiome composition: 2 related to stool dynamics—the time since the last bowel movement (bowel frequency) and the clock time of stool passage—and 2 additional factors: the time elapsed since the last sleep (sleep pressure) and time since the last meal (meal timing), both derived from the parent-completed 24-h diary entries.

### Characterization of Gut Microbiota

DNA was extracted from approximately 200 mg of stool using the PowerSoil kit, with minor modifications. After heat treatment (65 °C for 10 min, followed by 95 °C for 10 min), the samples were bead-beaten to lyse bacterial cells. The DNA extraction steps adhered to the manufacturer’s protocol. 16S rRNA gene amplicons targeting the V3 region were generated using specific primers compatible with the Nextera Index Kit (Illumina). Further details on amplification, barcoding, and sequencing are provided in Matenchuk et al. (2020) and Moore and Townsend (2019). A subsample of this data (SDEGU cohort) has been described in Schoch et al. (2022).

A comprehensive data processing and analysis pipeline was employed to identify zero-radius Operational Taxonomic Units (zOTUs) using UNOISE (Edgar and Flyvbjerg, 2015). Denoising and taxonomic assignment of amplicons were performed using USEARCH::UNOISE3 and USEARCH::Sintax with the SILVA SSU (v138) database. To improve data quality, samples with “time since last stool” greater than 48 h were excluded. Data normalization was performed through rarefaction to standardize sequencing depth across samples. Negative controls identified a potential contaminant (zOTU), but this had minimal effect on the final results. Core bacteria were defined as those present in at least 20% of samples with a minimum relative abundance of 1% (Heppner et al., 2024).

Three key metrics were used to assess the gut microbiome: diversity, evenness, and richness. Diversity (Shannon index) reflects the variety of species in a sample, with higher values representing a more diverse community. Evenness reflects how uniformly individual species are distributed, with higher evenness indicating a more balanced community. Richness (number of taxa) measures the total number of distinct species (Young and Schmidt, 2008). The relative abundance of zOTUs was also calculated and analyzed in relation to the 4 time-based factors (time since last stool, stool timing, sleep pressure, time since last meal). Abundance was determined by counting zOTUs that exceeded a relative abundance threshold of 0.01, identifying the key contributors to overall microbial diversity in each sample. In addition, the study examined the relative abundance of bacterial phyla—*Proteobacteria*, *Actinobacteria*, *Firmicutes*, and *Bacteroidetes*—focusing on the effects of time-related factors, across the age groups.

### Statistical Analysis

Linear regression analysis was conducted to assess relationships between microbial parameters and stool dynamics. Regression models estimated the association of independent variables, that is, time since the last bowel movement and the clock time of stool sampling, with microbial metrics, including diversity, evenness, and richness. The beta coefficient for each predictor variable was derived from the model to quantify the strength and direction of these associations. Age was included as covariates in the models to control for potential confounding effects. For repeated measures, linear mixed-effects models were employed, with random intercepts to account for within-subject variability. Correlation analysis was utilized to further explore the strength of these relationships. ANOVA was used to validate the findings from correlation analysis and to determine differences in microbial parameters based on variations in independent variables. Both linear regression and Pearson correlation were used to investigate the associations between microbial parameters and stool dynamics across 3 age groups. In addition, these statistical tests were used to examine the effects of stool dynamics on the composition of bacterial phyla. In addition to linear regression and mixed-effects models, we applied generalized additive models (GAMs) to better capture potential non-linear or rhythmic temporal dynamics in microbial parameters. GAMs were fitted with smoothing terms for each time-based variable (time since last stool, stool timing, sleep pressure, fasting duration), controlling for age group as a covariate.

## Results

### Time Since an Infant’s Last Bowel Movement Affects Gut Microbiome Composition

First, we examined the association between the time since the last bowel movement and the composition of the infant gut microbiota. Samples were grouped into 3-h intervals based on the time since the last stool (ranging from 1 to 48 h, with samples outside this range excluded, that is, 20 samples) to reduce noise and variability in the data. For example, samples collected within 1 to 3 h after a bowel movement were grouped into the 3-h time block, while those collected 4 to 6 h afterward were represented in the 6-h block. This method ensured a balanced representation of samples across time intervals.

The summary of microbial diversity, richness, and evenness across different time intervals since the last stool indicates that the mean Shannon diversity increases as the time interval progresses, peaking at 45 h (mea*n* = 2.67), with variability in standard deviation (SD) across intervals (ranging from 0.27 to 1.51). The richness values show a similar upward trend, with the highest mean richness observed at 42 h (mea*n* = 487.50), followed by gradual fluctuations, while the SD for richness is consistently higher than that for Shannon diversity. The mean evenness ranges from 0.38 to 0.53, with values generally increasing as the time intervals grow. The sample sizes vary across intervals, with the largest sample size at 24 h (99 samples) and smaller sizes at extreme intervals (e.g., 2 samples at 42 and 45 h) (Supplementary Table S1).

**Figure 1.**
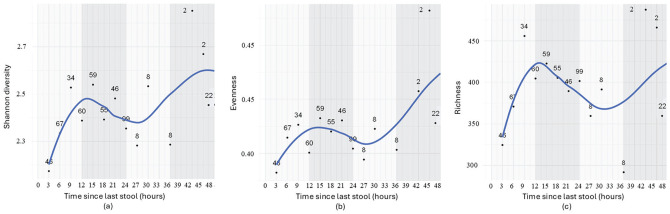
Relationship between time since the last bowel movement and gut microbial diversity (a), evenness (b), and richness (c) in infants (*n* = 504 samples: *n* = 152 at 3 months, 174 at 6 months, 137 at 12 months, and *n* = 41 at 5-31 months). Samples were grouped into 3-h intervals to adjust for uneven representation of sample numbers across time points. For each interval, averages of microbial parameters (diversity, evenness, and richness) were calculated. The displayed numbers indicate the number of samples included in each interval. The blue line represents the actual changes in microbiome parameters as time since the last stool changes, while the numbers in plots represent the number of samples per interval. Alternating light and dark gray bands denote 12-h periods for better visual orientation.

**Figure 2.**
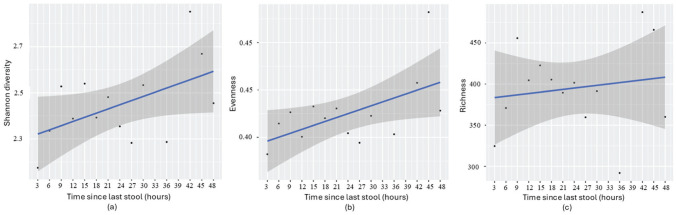
Regression analysis examining the relationship between time since the last stool and (a) Shannon diversity (*p* = 0.056, β = 0.018), (b) evenness (*p* = 0.038, β = 0.004), and (c) richness (*p* = 0.60, β = 1.656) in infants. Samples were grouped into 3-h intervals, consistent with Figure 1.

Within each time block, data of samples were averaged for key microbial parameters, including diversity, evenness, and richness (Figures 1 and 2). As shown in Figure 1, variations in time since the last stool related to microbial parameters, specifically, we observed an increase in diversity, evenness, and richness across the first 12 h, followed by a slight decrease between approximately 12 and 30 h, and a subsequent increase after approximately 30-36 h without stool passage, yet the later with increasing variance between individual time blocks.

Linear regression analysis revealed that longer intervals since the last bowel movement were associated with increased microbial diversity (Shannon *p* = 0.056 and β = 0.018) and evenness (*p* = 0.038, Beta β = 0.004), suggesting a more diverse and balanced microbial community over time (Figure 2). However, microbial richness did not show a significant association in the linear model (*p* = 0.605, β = 1.656). Together, these results suggest that while microbial richness remains relatively stable, a gradual enrichment may occur over time, consistent with the persistence of a core microbial community that slowly diversifies between bowel movements. In alignment, correlation coefficients showed strong positive relationships between time since the last stool and both microbial diversity (*r* = 0.51, *p* = 0.056) and evenness (*r* = 0.56, *p* = 0.038), without such effect for richness (*r* = 0.15, *p* = 0.605).

To further examine the relationship between stool frequency and microbial parameters, a 1-way ANOVA was conducted. The results were consistent with the findings from the regression analysis. Time since the last stool was significantly associated with Shannon diversity (*F* = 4.23, *p* = 0.056) and evenness (*F* = 5.43, *p* = 0.038; 3-h intervals), indicating that longer intervals between bowel movements are linked to increased microbial diversity and a more even distribution of species. Again, no association was observed for richness (*F* = 0.29, *p* = 0.601), suggesting that while time since last stool explains variability of microbial diversity and evenness, it does not explain the dynamics in the overall number of microbial taxa present.

**Figure 3.**
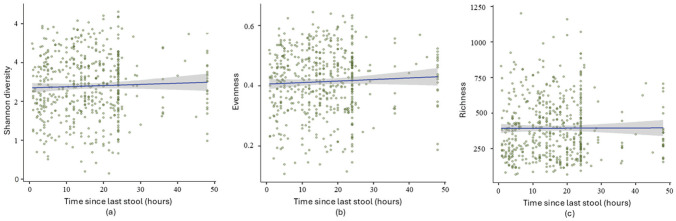
Mixed-effects model analysis of (a) Shannon diversity (*p* = 0.025, coeff = 0.065), (b) evenness (*p* = 0.031, coeff = 0.53), and (c) richness (*p* = 0.090, coeff = 0.071) in relation to time since the last stool in infants. All samples were analyzed individually without grouping into time intervals.

Next, Linear mixed-effects-models (LMM) were implemented with participant (infant) included as a random effect to account for repeated measures, and age treated as a fixed covariate to adjust for potential confounding.The analysis was used on all samples (without interval grouping) to capture variance between infants across continuous time points. This model accounts for repeated measures within the same infants across different age groups, following an approach similar to MaAsLin2 for handling longitudinal data. Regression coefficients (coeff) and *p*-values (*p*) were computed. The analysis revealed that longer intervals since the last bowel movement were associated with increased microbial diversity (Shannon: coef = 0.065, *p* = 0.025), evenness (coef = 0.053, *p* = 0.03), and richness (coef = 0.072, *p* = 0.009), suggesting that microbial communities become more diverse and balanced over time (Figure 3). Although these associations were statistically significant, with modest the effect sizes, indicating that longer intervals between bowel movements contribute moderately to variations in microbial diversity and evenness.

Results from grouped samples (Figures 1 and 2) and ungrouped samples (Figure 3) confirmed significant associations between microbial diversity and evenness with time since the last stool. Richness was only significant in the ungrouped analysis, likely due to preserved individual variability.

Finally, GAM analysis confirmed that time since last stool was significantly associated with microbial composition. The smooth term for time since last stool was significant (edf = 1, *F* = 6.81, *p* = 0.009), indicating a positive, though modest, non-linear trend. The model explained 31.6% of the variance (adjusted *R*^2^ = 0.31). This supports the findings from the linear regression and suggests that longer intervals between bowel movements are associated with increased microbial diversity. Richness was also positively associated with time since last stool (edf = 1, *F* = 4.39, *p* = 0.037). The effect was modest but significant, with the model explaining 44.1% of the variance (adjusted *R*^2^ = 0.44). Evenness showed a significant positive association with time since last stool (edf = 1, *F* = 5.48, *p* = 0.020). The effect was weaker compared to diversity and richness, with 18.7% of the variance explained (adjusted *R*^2^ = 0.18).

### Infants Stool Timing Affects Gut Microbial Diversity and Evenness

**Figure 4.**
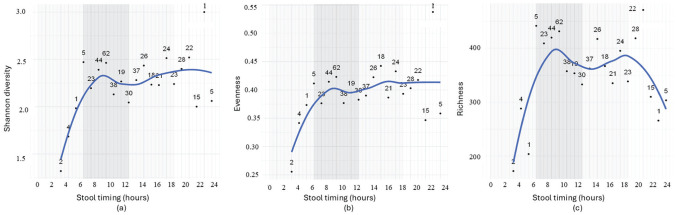
The association between stool timing and gut microbial diversity (a), evenness (b), and richness (c) in infants. The infant samples are grouped into 1-h intervals, and all samples in the same interval was averaged in terms of gut microbiome parameters. Alternating light and dark gray bands denote 6-h periods to facilitate visual orientation.

**Figure 5.**
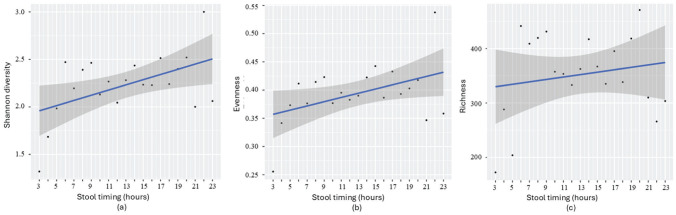
Regression analysis of (a) Shannon diversity (*p* = 0.056, β = 0.018), (b) evenness (*p* = 0.007, β = 0.003), and (c) richness (*p* = 0.53, β = 1.76) in relation to infant stool timing (1-24 h). Samples were grouped into 1-h intervals.

We next investigated the association between the timing of bowel movements (i.e., the clock time when the stool sample was obtained) and microbial parameters. Similarly, samples within close time frames were grouped into 1-h intervals, and the corresponding gut microbial parameters were averaged (Figures 4 and 5). The trajectories indicate, from a descriptive perspective, that variations in stool timing throughout the 24-h day—with data obtained within the range 3 am to 11 pm—relate to variance in microbial parameters. Specifically, diversity and evenness increased across the second half of the night from approximately 3 am to 10 am, followed by a period of stability across the daytime until approximately 11 pm. Meanwhile, richness showed a similar pattern first—increasing approximately from 3 am to 10 am, stabilizing between 10 am and 7 pm—but then starting to decrease toward midnight. Overall, these patterns reveal the notable observation that infant gut microbial parameters exhibit the greatest fluctuation during nighttime hours.

The summary of microbial diversity, richness, and evenness across 1-h stool timing intervals indicates that Shannon diversity generally increases with time, with some fluctuations, peaking at 22 h (mea*n* = 3.00, based on a single sample). Richness shows variability across intervals, with a high mean richness observed at 6 h (441.60) and 20 h (443.81). Evenness is relatively consistent across intervals, ranging from 0.26 to 0.54. The standard deviation (SD) highlights variability within each interval, and sample sizes vary widely, with some intervals having very few samples (e.g., 1 sample at 5 and 22 h) (Supplementary Table S2).

Linear regression analysis of 1-h interval samples confirmed the dynamic patterns by demonstrating a significant increase in diversity (*p* = 0.045, β = 0.024) and evenness (*p* = 0.07, β = 0.003) with later stool timing, as assessed within the time frame 3 am to 11 pm. Richness, however, did not show a significant relationship with stool timing (*p* = 0.53, β = 1.76). Correlation analysis revealed coefficients *r* = 0.50 (*p* = 0.021) for diversity, *r* = 0.45 (*p* = 0.043) for evenness, and *r* = 0.17 (*p* = 0.434) for richness. ANOVA was conducted to further explore the relationship between stool timing and gut microbiome parameters. The analysis confirmed that stool timing affects microbial parameters, with Shannon diversity (*F* = 6.383, *p* = 0.020), and evenness (*F* = 4.708, *p* = 0.042). However, no significant effect was observed for richness (*F* = 0.637, *p* = 0.435), indicating that stool timing does not affect the overall number of microbial species present.

LMM were applied to all samples continuously (without interval grouping). No significant associations were found between stool timing and gut microbiome parameters, except for a weak negative association with richness (coef = −0.041, *p* = 0.09, Supplementary Fig. S2). Increased variability and age adjustment likely masked trends observed in grouped data.

Finally, GAM analyses indicated that stool timing was not significantly associated with microbial diversity (*p* = 0.36) or evenness (*p* = 0.11), while richness showed only a weak, near-significant trend (*p* = 0.054). Variance explained by the models ranged from 17.5% to 43.8%, primarily driven by age effects, suggesting that diurnal influences on microbial community structure were modest compared with defecation interval and sleep pressure.

### Sleep Pressure and Fasting Periods Determine Infant Gut Microbiome Parameters

We then investigated whether variations in infant gut microbiome composition are associated with the duration of prior wakefulness (sleep pressure) and fasting (time since last meal). This analysis was conducted using 504 samples across all cohorts. Samples were grouped into 1-h intervals, and the gut microbial parameters (diversity, evenness, richness) were averaged within each 1-h interval.

**Figure 6.**
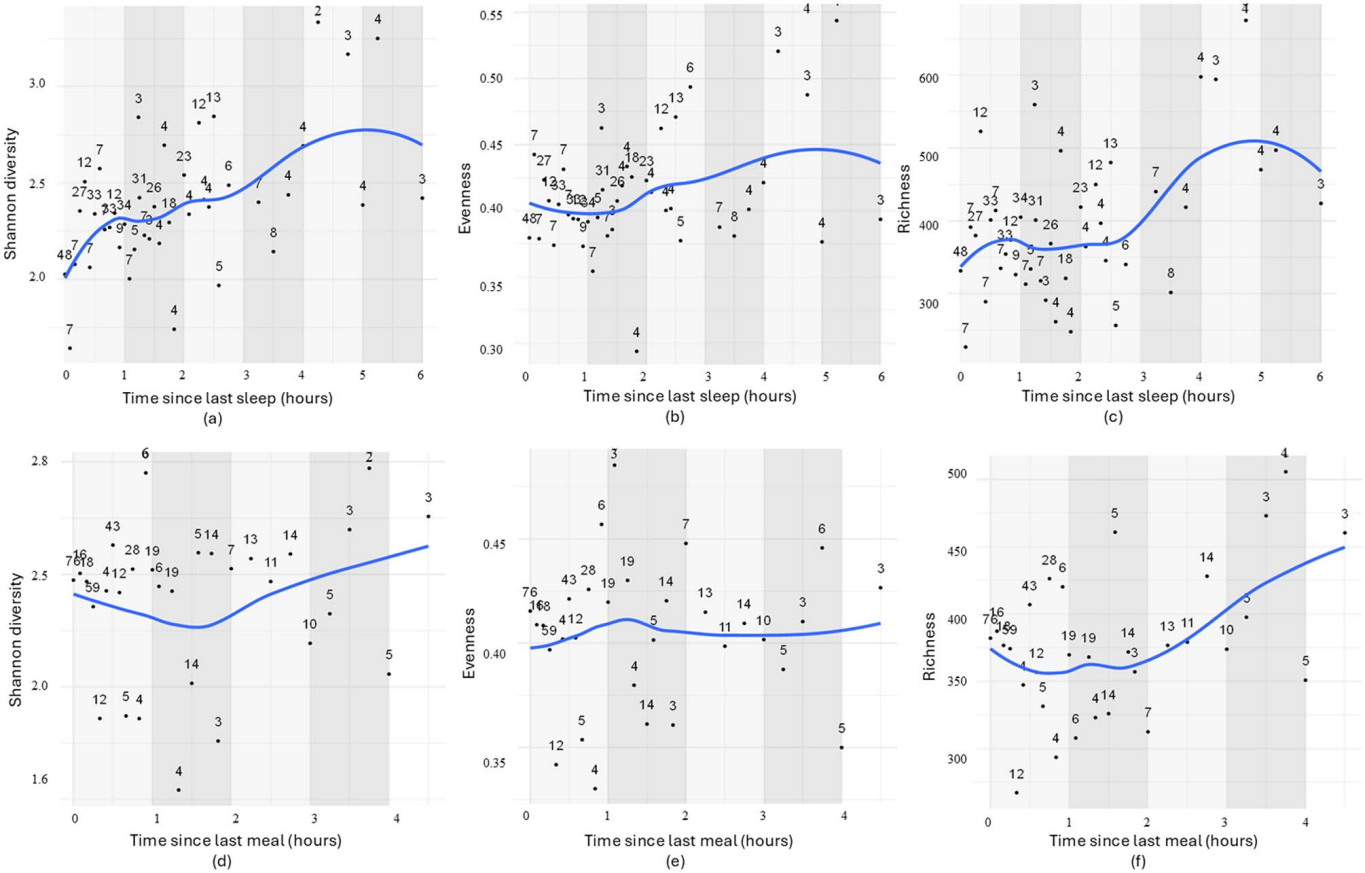
Relationship between sleep pressure (time since last sleep) and time since last meal with gut microbial diversity (a), evenness (b), and richness (c), and time since last meal with gut microbial diversity (d), evenness (e), and richness (f). All samples within a 15-min time interval were grouped and averaged for gut microbial parameters (diversity, evenness, richness).

Samples within up to 6 h after the last sleep episode were included (exclusion of 9 outlier samples). Sleep pressure was significantly associated with all microbial dynamics: Longer wakefulness before sampling was linked to increased diversity (*p* = 0.0002, *r* = 0.54), richness (*p* = 0.001, *r* = 0.48) and evenness (*p* = 0.023, *r* = 0.35), as shown in Table 3. Detailed time trajectories suggest an immediate and rapid increase in diversity, a delayed rise in richness, beginning approximately 3 h later, and a gradual increase in evenness (Figure 6a-c).

**Table 3.** Regression (*p*-values p) and correlation coefficients (*r*) of the associations among sleep pressure (time since last sleep) and time since last meal with microbial composition parameters.

Variables	Sleep Pressure		Time Since Last Meal	
	*p*	*r*	*p*	*r*
Diversity	**0.0002**	**0.54**	0.280	0.20
Richness	**0.001**	**0.48**	**0.009**	**0.47**
Evenness	**0.023**	**0.35**	0.738	0.06

Most samples were collected within the first 2 h after the last meal. With longer fasting, only bacterial richness displayed a statistically significant increase (Table 2). In line with this, detailed time trajectories suggest minimal dynamics in diversity and evenness in the immediate aftermath of a meal or fasting. However, the increase in richness becomes more apparent after 2 h and beyond (Figure 6d-f).

Finally, GAM analysis showed no significant associations between sleep pressure (time since last sleep) and microbial diversity, richness, or evenness, indicating that, unlike defecation interval, sleep pressure did not exhibit detectable non-linear effects on microbial community structure. Similarly, no significant associations were found between time since last meal and microbial diversity, richness, or evenness, suggesting that fasting duration did not exert detectable non-linear effects on gut microbiome composition.

### Age-related Differences in Gut Microbiome Parameters in Relation to Timing Factors

To investigate how age influences the relationship between timing factors and gut microbiome composition, we analyzed samples from infants at 3, 6, and 12 months, that is, cohorts SDEGU *n* = 387, SPIN *n* = 43, and NUTR *n* = 31. Associations were examined separately for each age group to explore developmental patterns in stool dynamics, sleep pressure, and time since last meal.

#### Age-related Differences in Stool Patterns and Gut Microbiome Parameters

We first examined how age may influence the relationship between stool timing and gut microbiome parameters, by focusing on longitudinal changes within-subject sampling (Table 4 and Supplementary Fig. S1). The infant samples are grouped into 3-h intervals in time since last stool and 1-h intervals in stool timing variable. We examined the association of stool timing and microbiome composition separately for each age group with liner regression analysis (Table 4).

**Table 4.** Regression analysis between stool timing parameters and microbial parameters represented for each age group (*p*-values p; Pearson correlation r).

	(a) Time Since Last Stool						(b) Stool Timing					
	3 Months		6 Months		12 Months		3 Months		6 Months		12 Months	
	*p*	*r*	*p*	*r*	*P*	*r*	*p*	*r*	*p*	*r*	*p*	*r*
Diversity	**0.021**	**0.66**	**0.033**	**0.59**	0.554	0.21	**0.019**	**0.52**	0.224	0.27	0.179	-0.34
Richness	0.223	0.46	0.368	0.2993	0.229	0.42	0.079	0.42	0.609	0.11	0.067	-0.45
Evenness	**0.035**	**0.57**	0.156	0.3992	0.653	0.16	0.084	0.38	0.454	0.14	0.228	-0.31

Time passed since last bowel movement was a significant factor explaining microbial variance at age 3 months, showing a positive linkage with microbial diversity and evenness. At 6 months, this effect prevailed with microbial diversity, yet by 12 months, no significant associations were observed. These data reveal strong effects of bowel frequency on microbial composition at the youngest age, and a gradual vanishing across the first year of life.

Similar relationships were observed for stool timing (clock stool): At 3 months, later stool timing was linked to increased microbial diversity and trends prevailed for evenness and richness. At 6 months, effects disappeared, and by 12 months, notably, richness revealed a negative association reaching trend-level significance.

**Figure 7.**
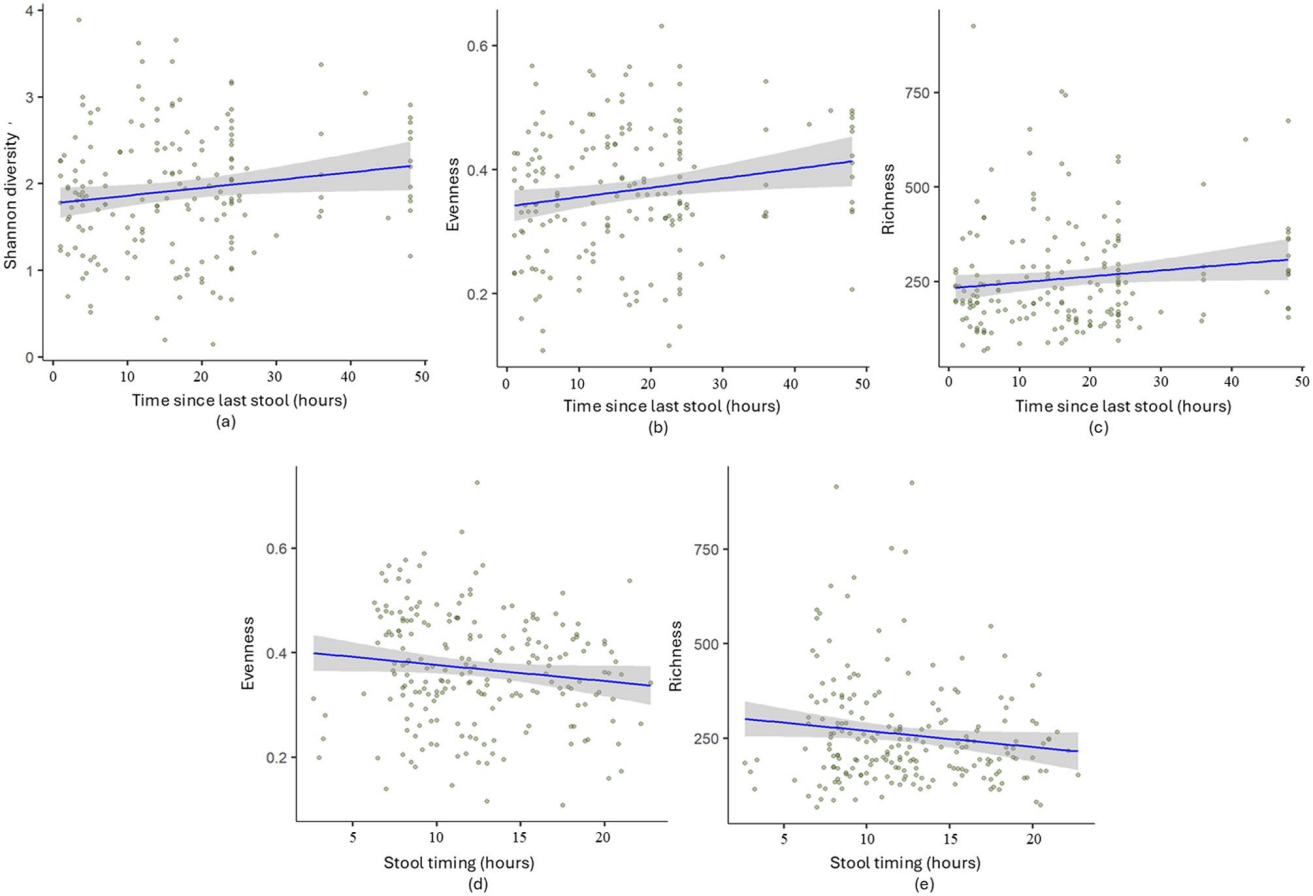
Stool dynamics and gut microbiome parameters at age 3 months, using all samples continuously (without interval grouping). Panels (a-c) show associations between time since last stool and gut microbial diversity (*p* = 0.081, coeff = 0.09), evenness (*p* = 0.022, coeff = 0.079), and richness (*p* = 0.016, coeff = 0.12), respectively. Panels (d-e) display associations between stool timing and gut microbial evenness (*p* = 0.012, coeff = –0.05) and richness (*p* = 0.14, coeff = –0.071).

Next, the LMM analysis was applied to infant samples individually (without interval grouping) across 3 infant age groups. Significant associations were observed at 3 months between gut microbiome parameters and time since the last stool (Figure 7a-c), as well as weak links between stool timing and gut microbiome parameters (Figure 6d-e). At 3 months (Figure 7a-c), time since the last stool was linked to increased microbial diversity, with trends also observed for richness and evenness. Stool timing at 3 months was weakly linked to richness and evenness but showed no association with diversity. At 6 and 12 months, no significant associations were found with any gut microbiome parameters. The results from ungrouped infant samples based on time intervals support the findings from grouped samples discussed in Table 4.

#### Age-related Differences in Sleep Pressure and Time Since Last Meal

Next, we assessed the relationships between sleep pressure and time since last meal with gut microbial parameters at different ages. Indeed, age-related effects were observed in sleep pressure and microbial diversity: We observed associations of sleep pressure with microbial diversity at 3 months (trend-level) and 6 months, but not 12 months. Neither effects for richness nor for evenness were observed at any age (Table 5). In other words, data indicate that long wakeful periods relate to a more diverse internal microbial landscape in young infants until 6 months but not beyond this age.

**Table 5.** Linear regression *p*-values (*p*) and correlation (*r*): association between time-based factors (time since last sleep, and time since last meal) and infant microbiome composition.

Variable	Sleep Pressure						Time Since Last Meal					
Age (Months)	3		6		12		3		6		12	
Measure	*p*	*r*	*p*	*r*	*p*	*r*	*p*	*r*	*p*	*r*	*p*	*r*
Diversity	**0.051**	**0.41**	**0.023**	**0.42**	0.662	0.09	0.329	0.22	0.419	0.17	0.673	0.10
Richness	0.469	0.16	0.546	0.23	0.872	0.03	0.310	0.23	0.773	0.06	0.230	0.28
Evenness	0.664	0.10	0.227	0.12	0.737	0.07	0.199	0.28	0.925	-0.02	0.657	0.11

Analyses of time since last meal unraveled that the above noted effect of fasting time on microbial richness in the total sample with combined age groups disappeared when analyzing by age group, identifying age as a confounder in the effect fasting may elicit on microbial richness.

Next, LMM analysis revealed no significant associations between sleep pressure and microbiome parameters. Shannon Diversity showed a weak association (β = 0.03, *p* = 0.13), while richness (β = −3.08, *p* = 0.365) and evenness (β = 0.001, *p* = 0.497) were non-significant. Similarly, “time since last meal” exhibited no significant associations, with weak trends for Shannon Diversity (β = −0.02, *p* = 0.504), richness (β = −6.73, *p* = 0.358), and evenness (β = <−0.00, *p* = 0.749). ANOVA revealed significant associations between age and sleep pressure on diversity (*F* = 167), evenness (*F* = 76.25), and richness (*F* = 272), as well as between age and time since last meal on diversity (*F* = 25), evenness (*F* = 76), and richness (*F* = 88.4) (*p* < 0.0001 for all), highlighting substantial developmental changes in the gut microbiome between 3, 6, and 12 months.

We extended our analysis to evaluate associations between gut microbiota diversity and timing variables at the individual sample level. While the interval-based grouping revealed several associations, these largely diminished when analyzed ungrouped. Across all ages, weak associations were observed between sleep pressure and both Shannon diversity (*p* = 0.076) and evenness (*p* = 0.068). At 3 months, sleep pressure showed a significant association with evenness (*p* = 0.038) and a marginal association with Shannon diversity (*p* = 0.08). No associations were found at 6 months, while a negative significant link between sleep pressure and microbial diversity re-emerged at 12 months (*p* = 0.034). Regarding feeding history, only non-significant negative associations with Shannon diversity (*p* = 0.06) and evenness (*p* = 0.062) were detected at 12 months. These findings suggest that while grouping by timing intervals may reveal general trends, inter-individual variability can attenuate these associations at the single-sample level.

### Beta Diversity and Compositional Differences Across Timing Factors and Age

To assess whether gut microbial community composition varied with timing-related factors and age, we performed β-diversity analyses using Bray–Curtis dissimilarity and permutational multivariate analysis of variance (PERMANOVA). Infant age significantly affected overall microbiome composition (*R*^2^ = 0.057, *F* = 25.91, *p* = 0.001), explaining 5.7% of the total variance (Table 6). A weak association was observed for sleep pressure (*R*^2^ = 0.0035, *F* = 1.51, *p* = 0.046), suggesting subtle compositional changes with prolonged wakefulness. In contrast, stool timing, time since last stool, and time since last meal were not significantly associated with overall microbial community structure. These findings indicate age as strong driver of compositional differences, while short-term temporal factors contribute only minimally to between-sample variation.

**Table 6.** PERMANOVA results assessing the effects of timing variables and age on gut microbial community composition (Bray–Curtis dissimilarity).

Variable	Sum of Squares	*R* ^2^	*F*	*p*-Value
Stool timing	0.377761	0.002338	1.000784	0.359
Time since last stool	0.373717	0.002313	0.990045	0.381
Time since last meal	0.47587	0.002946	1.261467	0.165
Sleep pressure	0.568724	0.00352	1.50848	**0.046**
Timepoint (3, 6, 12 months)	9.242595	0.05721	25.91106	**0.001**

Values *p* < 0.1 are indicated in bold.

### Effect of Feeding Type on Gut Microbiome Diversity and Temporal Patterns

How did different feeding methods influence infant gut microbiome development and daily physiological rhythms? Linear mixed-effects models were used to assess these associations.

#### Feeding Type and Gut Microbiome Diversity

Infant feeding type significantly determined gut microbiome development, affecting all diversity metrics (Supplementary Table S2a, Supplementary Fig. S3). Formula-fed infants showed substantially richer (+70.7 units, *p* = 0.0005) and more diverse (+0.33 units, *p* = 0.0004) bacterial communities compared to breast-fed infants, while mixed-fed infants showed moderate but significant increases in both richness (+32.3 units, *p* = 0.021) and diversity (+0.18 units, *p* = 0.006). The same pattern held for microbial evenness, which increased with both formula (+0.039 units, *p* = 0.006) and mixed feeding (+0.027 units, *p* = 0.006). As shown in Figure 3, this pattern remained consistent across all 3 age points (3, 6, and 12 months), with formula feeding consistently associated with the highest diversity values, followed by mixed feeding, and then breastfeeding. In addition, age independently contributed to all diversity metrics, with each additional month associated with increased richness, diversity, and evenness (*p*-values < 0.0001).

#### Feeding Patterns and Temporal Factors

Feeding method affected infant sleep patterns but not digestive rhythms (Supplementary Table 3b). Formula-fed infants had significantly longer time intervals since last sleep (+0.42 h, *p* = 0.015) to stool sampling, compared to breast-fed infants, equivalent to approximately 25 min longer to build up sleep pressure. Mixed feeding showed intermediate sleep patterns (+0.14 h, *p* = 0.350) that were not statistically significant.

No associations emerged between feeding type and other temporal measures (Supplementary Table 3b). Mixed feeding showed a borderline trend for longer stool intervals (+2.1 h, *p* = 0.08) while all other comparisons for meal timing, stool timing, and bowel movement clock time were non-significant.

### Associations Between Time-Based Factors and zOTUs Relative Abundance and Bacterial Phyla

**Figure 8.**
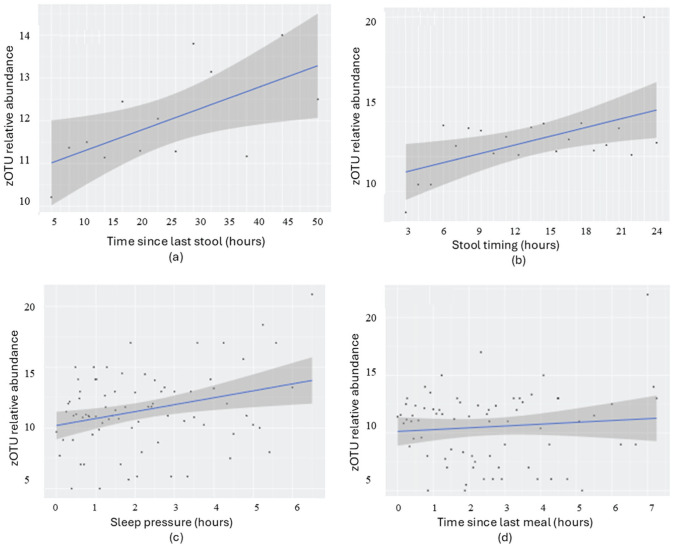
Regression analysis of infant group samples with relative abundances > 0.01, examining associations with temporal factors: (a) Time since last stool (*p* = 0.023), (b) Stool clock time (*p* = 0.014), (c) Sleep pressure (*p* = 0.008), and (d) Time since last meal (*p* = 0.425). Samples (*n* = 504, including all ages and cohorts) were grouped into time intervals: 3-h intervals for (a), 2-h intervals for (b), and 1-h intervals for (c) and (d). This threshold 0.01 was applied to focus on biologically relevant, high-abundance taxa while minimizing sequencing noise.

We then refined the analysis by incorporating the relative abundance of zOTUs to achieve a more precise understanding of underpinnings of microbial diversity. Abundance was measured by counting active zOTUs (i.e., that exceeded a relative threshold of 0.01), focusing on the key contributors in each sample. Positive relationships between zOTU relative abundance and time since last stool (*p* = 0.023, *r* = 0.62), stool sampling time (*p* = 0.014, *r* = 0.53), and sleep pressure (*p* = 0.008, *r* = 0.36) were observed, while time since the last meal showed no significant effect (*p* = 0.45, *r* = 0.1; Figure 8).

To validate these findings at the individual sample level, we extended the analysis and confirmed a robust association between abundance and time since last stool (*p* = 0.004). A marginal relationship was also observed with sleep pressure (*p* = 0.07), further supporting the influence of physiological and behavioral timing cues on microbial composition, while stool timing and meal timing remained non-significant.

### Timing Effects on Major Gut Bacterial Phyla in Infants

**Figure 9.**
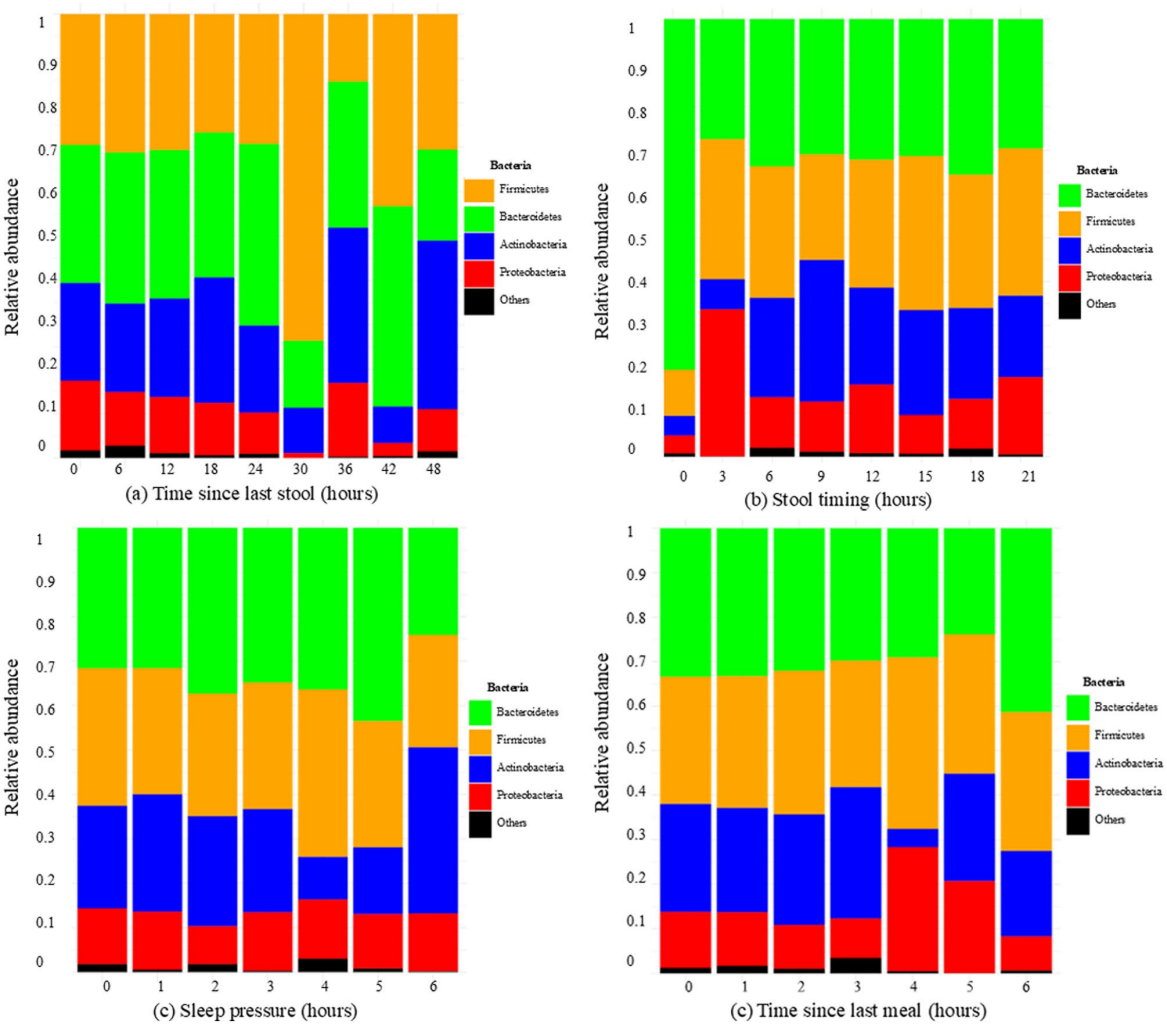
Relative abundance of major bacterial phyla in the infant gut by (a) time since last stool, (b) stool timing, (c) sleep pressure, and (d) time since last meal. Samples are grouped into 6-, 3-, 1-, and 1-h intervals for l, respectively, to adjust for unevenly represented hours along the x-axis.

Next, we extended the analysis to the relative abundance of the major bacterial phyla—*Proteobacteria*, *Actinobacteria*, *Firmicutes*, and *Bacteroidetes*. The analysis included all samples across ages (*n* = 504; 152 at 3 months, 174 at 6 months, 137 at 12 months, and 41 between 5 and 31 months). Linear regression analyses were performed, with correlation coefficients (*r*) reported to indicate the strength and direction of associations. The time since the last stool related negatively with *Proteobacteria* abundance (*p* = 0.027, *r* = −0.15), indicating lower *Proteobacteria* levels with lower stool frequency. Conversely, Actinobacteria showed a modest positive correlation with time since last stool (*p* = 0.056, *r* = 0.13), indicating higher levels with lower stool frequency. Among clock time, sleep pressure, and time since last meal no relationship was detected at the phyla level (Figure 9 and Table 7).

**Table 7.** Regression *p*-values (*p*) and correlation (*r*): relative abundance of bacterial phyla in the infant gut by time-related factors across 3 age groups (3, 6, and 12 months).

Variable	(a) Time Since Last Stool						(b) Stool Timing					
Bacterial Phyla	3		6		12		3		6		12	
	*p*	*r*	*p*	*r*	*p*	*r*	*p*	*r*	*p*	*r*	*p*	*r*
***Actinobacteria***	0.341	0.10	0.978	-0.01	**0.003**	**0.29**	0.161	-0.14	**0.069**	**0.18**	0.635	-0.04
***Bacteroidetes***	0.563	-0.06	0.507	0.07	0.639	-0.04	0.419	0.08	0.635	-0.04	0.247	-0.11
***Proteobacteria***	**0.017**	**-0.24**	0.880	0.01	**0.071**	**-0.18**	0.245	-0.11	0.299	-0.10	0.383	0.08
***Firmicutes***	0.813	0.10	0.343	-0.10	0.300	-0.10	0.245	0.11	0.577	-0.05	0.244	0.11
Others	0.437	-0.08	0.821	-0.02	0.469	-0.07	0.251	0.11	0.167	-0.13	0.455	0.07
Variable	(c) Sleep Pressure						(d) Time Since Last Meal					
Bacterial Phyla	3		6		12		3		6		12	
	*p*	*r*	*p*	*r*	*p*	*r*	*p*	*r*	*p*	*r*	*p*	*r*
***Actinobacteria***	0.705	0.04	0.649	0.04	0.496	-0.07	0.981	0.01	0.4	-0.08	0.541	0.06
***Bacteroidetes***	0.189	0.13	0.585	0.05	0.832	-0.02	0.158	-0.15	0.665	-0.04	0.574	0.05
***Proteobacteria***	0.786	0.03	0.499	-0.07	0.837	0.02	0.279	0.11	0.592	0.05	0.281	-0.11
***Firmicutes***	**0.052**	**-0.20**	0.535	-0.06	0.611	0.05	0.685	0.04	0.219	0.12	0.7	-0.04
Others	0.589	0.06	0.558	-0.06	0.114	0.16	0.312	0.10	0.692	0.04	0.334	-0.12

Values *p* < 0.1 are indicated in bold.

To further differentiate temporal effects on microbiome maturation, we conducted analyses in relation to age timepoints (3, 6, 12 months). Primarily bowel frequency was a relevant determinant: lower stool frequency was linked to decreased *Proteobacteria* at 3 and 12 months (the later at trend-level; Table 7). At 12 months, lower stool frequency was linked to increased *Actinobacteria*. Second, the timing of stool (clock stool) revealed some link to bacterial phyla, shown in a trend-level association with *Actinobacteria* at age 6 months only. Third, sleep pressure recorded an association with bacterial phyla relative abundance, concerning a decrease in *Firmicutes* with longer time wake at age 3 months. Finally, time since last meal did not exhibit any association with bacterial phyla relative abundance.

Overall, the analysis shows that effects of temporal factors on the gut microbiome vary considerably across infancy. This highlight age-specific transitions in the shaping of gut microbiota.

To control for multiple comparisons and reduce the likelihood of false positives, we applied false discovery rate (FDR) correction to our analyses of associations between the 4 time-based factors in Table 7, and the results are reported in Table 8. After FDR correction, only *Actinobacteria* at age 12 months remained statistically significant (*p* = 0.045, Table 8a), suggesting a robust association where less frequent stool is linked to increased *Actinobacteria* levels.

**Table 8.** Original *p*-values (*p*) and False Discovery Rate–corrected *p*-values (*FDR*) for bacterial phyla in the infant gut by time-related factors across 3 age groups (3, 6, and 12 months).

Variable	(a) Time Since Last Stool						(b) Stool Timing					
Bacterial Phyla	3		6		12		3		6		12	
	*p*	*FDR*	*p*	*FDR*	*p*	*FDR*	*p*	*FDR*	*p*	*FDR*	*p*	*FDR*
***Actinobacteria***	0.341	0.844	0.978	0.978	**0.003**	**0.045**	0.161	0.470	**0.069**	0.470	0.635	0.635
***Bacteroidetes***	0.563	0.844	0.507	0.844	0.639	0.871	0.419	0.568	0.635	0.635	0.247	0.470
***Proteobacteria***	**0.017**	0.127	0.880	0.942	**0.071**	0.355	0.245	0.470	0.299	0.498	0.383	0.568
***Firmicutes***	0.813	0.942	0.343	0.844	0.300	0.844	0.245	0.470	0.577	0.635	0.244	0.470
Others	0.437	0.844	0.821	0.942	0.469	0.844	0.251	0.470	0.167	0.470	0.455	0.568
Variable	(c) Sleep Pressure						(d) Time Since Last Meal					
Bacterial Phyla	3		6		12		3		6		12	
	*p*	*FDR*	*p*	*FDR*	*p*	*FDR*	*p*	*FDR*	*p*	*FDR*	*p*	*FDR*
***Actinobacteria***	0.705	0.837	0.649	0.837	0.496	0.837	0.981	0.981	0.4	0.750	0.541	0.750
***Bacteroidetes***	0.189	0.837	0.585	0.837	0.832	0.837	0.158	0.750	0.665	0.750	0.574	0.750
***Proteobacteria***	0.786	0.837	0.499	0.837	0.837	0.837	0.279	0.750	0.592	0.750	0.281	0.750
***Firmicutes***	**0.052**	0.780	0.535	0.837	0.611	0.837	0.685	0.750	0.219	0.750	0.7	0.750
Others	0.589	0.837	0.558	0.837	0.114	0.837	0.312	0.750	0.692	0.750	0.334	0.750

## Discussion

Understanding the interplay between time-based factors and gut microbiota composition in early childhood is critical for advancing our knowledge of microbial development, which this study sought to examine. We analyzed 509 stool samples from 198 healthy infants, examining their immediate context, including time since last stool, sleep pressure, meal history, and clock time of stooling. Our results unveil 5 new insights: (1) existence of a linkage between a gut microbial composition and bowel movement time, (2) evidence age-specific transition in how gut microbiome parameters are affected by temporal factors, (3) strong associations between sleep pressure and gut microbiome composition, (4) yet no linkage with fasting periods, and (5) associations of bowel movement frequency with zOTU abundance and bacterial phyla.

This comprehensive study unraveled an interplay between bowel movement frequency and various microbial parameters. Specifically, longer intervals since the last stool were associated with increased microbial diversity and evenness. These results align with existing research highlighting the infant gut microbiome’s sensitivity to bowel movement frequency (Agans et al., 2011; La Rosa et al., 2014). The observed rise in microbial diversity and evenness may reflect nutrient depletion prior defecation, indicating that the gut microbiome requires time to recover and re-establish diversity (De Filippo et al., 2010). We further report that zOTU relative abundance is significantly associated with temporal factors, reflecting the dynamic nature of the gut microbiome in response to physiological changes. A positive association between time since the last stool and zOTU abundance suggests a recovery period post-defecation, during which the gut environment stabilizes, promoting microbial colonization (David et al., 2014). Interestingly, the findings highlight that association between time since the last stool and gut diversity and evenness is high at younger infant ages and diminishes as infants mature. These findings suggest that the gut microbiome’s responsiveness to time-related stool factors evolves with age, highlighting a dynamic pattern of infant gut microbial development (Roager et al., 2023). Although statistically significant, the effect sizes were modest, suggesting that temporal dynamics account for only a small portion of microbial variance. However, even subtle yet consistent effects across cohorts may still hold biological relevance in early microbial development. In addition, GAM analyses confirmed that time since last stool was consistently associated with increased microbial diversity, richness, and evenness. While the effects were modest in magnitude, they were statistically significant and align with the hypothesis that longer intervals between bowel movements allow for greater microbial community development and balance. The approximately linear smooth terms suggest gradual rather than strongly oscillatory dynamics.

We observed an effect of stool timing (clock time) on microbial composition. This may reflect the build-up of the enteric diurnal rhythm in the infant gut microbiome, which has been suggested based on bacterial communities in formula- and breast-fed infants (Forbes et al., 2018; Heppner et al., 2024). A recent study (Heppner et al., 2024) compared bacterial communities in formula- and breast-fed infants, clock time–based assessments revealed this rhythm, yet without accounting for the timing of stool samples in relation to other potential zeitgebers, which may not fully capture drivers of microbial rhythmicity. Beyond clock times of stool collection, our study additionally included stooling history, dietary and sleep-related context; thus, providing a comprehensive picture of diurnal dynamics within an age-controlled manner. Specifically, this approach evidenced sleep history as strong driver of diurnal microbial dynamics, whereas the effect of prior fasting time was surprisingly negligible.

Our data reveal that the gut microbiota becomes more diverse and evenly distributed as the day progresses, indicated by increased bacterial diversity and evenness with later stool timings. This confirms diurnal dynamics in infant gut microbiota composition, as previously proposed based on data modeling (Trosvik et al., 2010). Increased microbial diversity and evenness with later stool timings were proposed to be affected by feeding patterns (Zhao et al., 2022), with morning defecation maintaining reduced diversity due to overnight fasting and lower microbial activity (Kaczmarek et al., 2017). Feeding-type analysis showed higher richness and diversity in formula- and mixed-fed infants, supporting that feeding mode and nutrient complexity contribute to these microbial shifts. Yet, our novel results unravel that microbial effects of meal timing are primarily driven by age. Furthermore, the link of gut microbiome composition with the stool timing is very clear with younger infants and disappear with infant maturation (Roager et al., 2023), suggesting that the early gut microbiome is particularly sensitive to timing dynamics (Sprockett et al., 2018). This aligns with feeding transitions in our cohort, where breastfeeding predominated at 3 months and gradually shifted to mixed and formula feeding, paralleling the rise in microbial richness and evenness.

The observed link between stool timing and zOTU abundance further points to emerging entero-diurnal (possibly circadian) rhythms. Lower microbial abundance in the morning is likely also intertwined with overnight fasting, with abundance increasing throughout the day as metabolic activity and food intake rise (Thaiss et al., 2014). As the day progresses, increasing metabolic activity and nutrient intake change the local microbial environment, likely leading to higher zOTU counts later in the day. Overall, the effect of stool timing on microbial composition is a crucial discovery that could inform ongoing initiatives applying microbial composition as markers for infant health and disease. For example, gut microbiome can predict risks of gastrointestinal disorders like necrotizing enterocolitis (Pammi et al., 2017), and knowledge of factors that fine-tune the variations in microbial diversity, like diurnal patterns, may improve precision in identifying infants at risk of dysbiosis (Nakandalage et al., 2023). It may also fundamentally support the development of targeted prebiotics and probiotics for infants and young children, helping to optimize gut health and, indirectly, developmental outcomes (Schoch et al., 2020, 2022).

Sleep is essential for facilitating key physiological processes such as growth, immune function, and neurodevelopment across the lifespan (Matenchuk et al., 2020). Insufficient sleep fundamentally disrupts these processes, negatively affecting metabolism and behavior, and thus increasing the risk of various diseases (Itani et al., 2017). Poor sleep in infancy and early childhood has been linked to long-term consequences, including obesity, asthma, or cognitive dysfunction (Halal et al., 2016; Kozyrskyj et al., 2009). In the current investigation, we report a strong effect of sleep pressure on gut microbiome composition during infancy. Specifically, we highlight a microbial diversity increase with longer wake periods, indicating that microbial diversity benefits from resumed physiological processes during the waking period (Goldberg et al., 1988). In addition, the results revealed that richness and evenness also rise after just a brief wakefulness duration. These findings highlight an effect of sleep-wake history on gut microbiome composition, which may connect to the gradual growth of microbial enteral clocks (Sharon et al., 2024; Wollmuth and Angert, 2023). We also detail insights into different age groups. Interestingly, the link of sleep pressure to diversity and richness at the age of 3 months emphasizes a potentially critical role of sleep in early microbiome establishment. Sleep influences metabolic and immune functions, which in turn shape the gut microbiome (Anderson et al., 2017; Goldberg et al., 1988). The reduced effect of sleep on microbial composition at older ages may reflect the microbiome’s stabilization and growing resilience as infants sleep patterns and the circadian regulators become more solid. In addition, the findings showed that zOTU abundance increases with sleep pressure, suggesting that the body’s metabolic state after waking supports this increase in abundance (Bowers et al., 2020). This post-waking surge in zOTUs could indicate that as metabolic processes resume, the gut environment becomes more suitable for microbial growth and diversity. Interestingly, GAM analysis showed no significant non-linear associations between sleep pressure and microbial parameters, suggesting that its effects are modest, largely linear, and age-dependent, in contrast to the stronger non-linear effects of defecation interval.

Finally, we determined the effect of meal history as a source of influence on gut microbiome composition. Time passed between meal and stool unraveled a positive correlation with gut microbial richness, suggesting that longer intervals between feeding promote a more diverse microbiome. This positive correlation reinforces the role of feeding timing in promoting microbial growth and diversity, highlighting that feeding patterns shape gut microbiota composition, in alignment with (Mühlematter et al., 2024; Ramos et al., 2025). However, when the analysis was conducted separately for different age groups, this effect of meal history on microbial composition was no longer observed, indicating that age is a significant confounding factor in this relationship. One reason for this lack of effect could be that most samples collected for time since last meal fall within the first and second hour, which lead to a biased presentation of data. Unlike prior studies that predominantly examined feeding type (e.g., breastfeeding versus formula feeding; Forbes et al., 2018) or diet composition in later stages of adulthood (Wan et al., 2019), our study highlights the important role of meal timing on early-life microbiota composition. Additional analysis confirmed that feeding type independently determined microbial diversity, with formula-fed infants showing greater richness and evenness even after adjusting for age. The observations underscore the importance of considering feeding intervals alongside feeding type in infancy, as these patterns not only affect analyses but may also play a critical role in shaping early microbiota development. In addition, the results showed no significant association between zOTU abundance and prolonged fasting, except for a weak negative correlation, underscoring the reliance of microbial diversity on nutrient availability (Sonnenburg and Sonnenburg, 2014). zOTU abundance also declines as nutrient levels drop over time, suggesting fewer species can thrive in the less nutrient-depleted environment.

In addition, our analysis revealed that temporal factors associate with microbiome composition at the phylum level, with age-specific patterns: lower stool frequency reduced *Proteobacteria* at 3 and 12 months, while *Actinobacteria* increased at 12 months, consistent with their early microbial succession. Sleep pressure reduced *Firmicutes* at 3 months, associating wake duration to microbiome composition. However, no consistent links were found for stool timing or time since last meal, suggesting that microbial responses to these factors may be more dynamic and age-dependent. In addition, consistent with previous studies, β-diversity analyses confirmed that infant age was the predominant factor shaping gut microbial composition, whereas behavioral timing variables exerted comparatively minor or transient effects. These findings suggest that while temporal physiology influences within-sample diversity, the overall community structure remains largely age-dependent. Feeding-type analysis further supported the observed age-related trends in microbial diversity. Formula- and mixed-fed infants showed higher richness, diversity, and evenness compared to those exclusively breastfed (Supplementary Table S3a, Figure 3). These findings mirror the natural transition in our cohort from predominant breastfeeding at 3 months to increasing mixed and formula feeding by 12 months (Table 1b). This pattern indicates that feeding contributes to microbial diversification during infancy and should be considered alongside temporal factors in future analyses.

A critical question arising from these findings concerns their clinical relevance. Specifically, this study explores whether microbial shifts such as increased alpha diversity with longer defecation intervals or later stool timing may ultimately confer tangible health benefits. Interpreting these changes requires an age-dependent perspective: In early infancy, a gradual rise in alpha diversity is often a sign of healthy microbiome development (Micenková et al., 2025) toward a more complex and resilient ecosystem. Such progression is linked to improved metabolic and immune development. The observed patterns are more consolidated bowel rhythms and diurnal regularity, which likely indicate a shift toward a more stable and protective gut state (Alderete et al., 2021). However, this interpretation is not absolute, as diversity itself is not always synonymous with health and depends on the nature of the expanding taxa (Williams et al., 2024). While our study identifies consistent associations, it cannot establish causality, for which experimental studies are needed (Zimmermann et al., 2025). From the current insights, we propose that supporting temporal regularity may foster a microbiome that is metabolically efficient and resilient, though future longitudinal studies linking these microbial dynamics to direct health outcomes are warranted.

Overall, this study unravels the responsiveness of the infant gut microbiome to daily physiological and behavioral changes. The strongest effects were observed in the interplay between bowel movement frequency and microbial parameters, where longer intervals since the last stool were linked to increased microbial diversity and evenness. Stool timing (clock time) also had a strong impact on microbial composition, with diversity and evenness tending to increase later in the day. In addition, sleep pressure played a critical role, particularly at 3 months, where longer wake periods were associated with increased microbial richness and evenness. In comparison, meal history had a comparatively weaker effect on microbial richness, evident mostly in the immediate post-feeding period. Notably, the effects of bowel movement frequency, stool timing, and sleep pressure were more pronounced in younger infants, emphasizing the unique sensitivity of the early gut microbiome to temporal dynamic factors. The emerging synchrony between infant gut microbial diurnality and host circadian rhythms may reflect a conserved developmental coordination mechanism. This work opens ground for evolutionary anthropology, such that the gut-sleep alignment could scaffold metabolic, immune, and neurobehavioral regulation during infancy, potentially enhancing survival and long-term fitness (Litichevskiy and Thaiss, 2022). These findings may inform future studies to explore whether deviations from synchrony confer quantifiable developmental costs, from which to then design supplemental treatments to optimize early microbiome development and long-term health. In addition, antibiotic exposure was rare in this cohort, with no reported use at 3 months and only a small proportion thereafter (Table 1a). The absence of detailed post-enrollment antibiotic data remains a limitation and can be investigated in future studies. Likewise, combining feeding type and antibiotic exposure in future analyses may clarify their joint effects on microbial maturation. Finally, the present study focused on phylum-level analyses to maintain consistency across heterogenous sequencing data (batches) and to ensure comparisons across sub-cohorts. While this approach captures major compositional trends, future work using latest unified references such as Greengenes2 (McDonald et al., 2024) could enable finer taxonomic resolution at the genus or amplicon sequence variant (ASV) level, offering even deeper insight into temporal microbial succession and age-dependent specificity.

## Conclusion

The study highlights the association between stool dynamics and temporal factors that act as zeitgebers for establishing sleep rhythm across the first months of human life. Samples from 198 healthy infants aged 3-31 months provided the fundament to test associations between time since last stool, stool timing, sleep pressure, and time since last meal with gut microbiome composition (e.g., alpha diversity, richness, evenness; abundance and phyla). The study identified a negligible effect of immediate infant fasting history on microbial composition, but 2 strong microbial determinants in bowel frequency and sleep pressure. Notably, age-specific transitions emerged as the strongest predictors, with the sleep-gut microbiome link being most prominent—and thus most vulnerable—at the earliest ages. Our findings emphasize the heightened responsiveness of the infant gut microbiome to environmental cues, particularly at 3 months of age. In addition, the findings highlight the need for crucial adjustments when using microbiota profiles as indicators of health and development.

We call for future research to consider the role of temporal factors when conceptualizing clinical applications related to infant microbial diagnostics and nutritional interventions. Longitudinal studies tracking the impact of temporal cues on the infant gut microbiome, with a focus on transitions to solid foods and regular sleep patterns, will be essential. In addition, investigating the establishment of circadian rhythms in diverse populations could provide a deeper understanding of microbiota-related health, further informing clinical practice and interventions. Finally, while this study focused on phylum-level bacterial composition, future studies should extend this approach to the genus level to capture more nuanced microbial shifts and their potential functional implications.

## Supplemental Material

sj-docx-1-jbr-10.1177_07487304251407313 – Supplemental material for Stool Dynamics and the Developing Gut Microbiome During Infancy Supplemental material, sj-docx-1-jbr-10.1177_07487304251407313 for Stool Dynamics and the Developing Gut Microbiome During Infancy by Mohammed Al-Andoli, Sarah Schoch, Andjela Markovic, Christophe Mühlematter, Matthieu Beaugrand, Oskar G. Jenni, Rabia Liamlahi, Jean-Claude Walser, Dennis Nielsen and Salome Kurth in Journal of Biological Rhythms
