# Supplementary material for: Stool Dynamics and the Developing Gut Microbiome During Infancy
Source: J Biol Rhythms. 2026 Jan 20;41(3):324–43. doi: 10.1177/07487304251407313 (PMC13103348; doi:10.1177/07487304251407313)
Supplement: sj-docx-1-jbr-10.1177_07487304251407313 – Supplemental material for Stool Dynamics and the Developing Gut Microbiome During Infancy [file sj-docx-1-jbr-10.1177_07487304251407313.docx]

**Supplementary Material**

| Supplementary Table 1. Summary statistics of microbial diversity, richness, and evenness across time intervals since last stool. | | | | | | | |
| --- | --- | --- | --- | --- | --- | --- | --- |
| Time Intervals | Samples-per-group | Mean Shannon | SD Shannon | Mean Richness | SD Richness | Mean Evenness | SD Evenness |
| 3 | 46 | 2.17 | 0.65 | 324.65 | 172.17 | 0.38 | 0.09 |
| 6 | 67 | 2.34 | 0.86 | 370.91 | 203.84 | 0.41 | 0.12 |
| 9 | 34 | 2.53 | 0.78 | 455.82 | 253.29 | 0.43 | 0.09 |
| 12 | 60 | 2.39 | 0.81 | 404.65 | 186.66 | 0.40 | 0.11 |
| 15 | 59 | 2.54 | 0.85 | 422.73 | 197.67 | 0.43 | 0.10 |
| 18 | 55 | 2.39 | 0.89 | 405.45 | 225.95 | 0.42 | 0.11 |
| 21 | 46 | 2.48 | 0.81 | 389.54 | 231.03 | 0.43 | 0.10 |
| 24 | 99 | 2.35 | 0.84 | 401.64 | 209.54 | 0.40 | 0.11 |
| 27 | 8 | 2.28 | 0.87 | 359.62 | 294.17 | 0.39 | 0.10 |
| 30 | 8 | 2.53 | 0.65 | 391.50 | 164.81 | 0.42 | 0.09 |
| 36 | 8 | 2.29 | 0.74 | 291.88 | 125.77 | 0.40 | 0.10 |
| 42 | 2 | 2.85 | 0.27 | 487.50 | 194.45 | 0.46 | 0.02 |
| Supplementary Table 2. Summary of microbial diversity, richness, and evenness across stool timing intervals. | | | | | | | |
| Time Intervals | Samples-per-group | Mean Shannon | SD Shannon | Mean Richness | SD Richness | Mean Evenness | SD Evenness |
| 3 | 2 | 1.32 | 0.43 | 172.5 | 16.26 | 0.26 | 0.08 |
| 4 | 4 | 1.69 | 0.49 | 288.25 | 167.64 | 0.34 | 0.11 |
| 5 | 1 | 1.98 | 0.51 | 204 | 170.8 | 0.37 | 0.10 |
| 6 | 5 | 2.47 | 0.89 | 441.6 | 374.55 | 0.41 | 0.09 |
| 7 | 32 | 2.39 | 0.68 | 422.25 | 158.48 | 0.41 | 0.1 |
| 8 | 61 | 2.48 | 0.9 | 415.13 | 217.56 | 0.43 | 0.11 |
| 9 | 76 | 2.53 | 0.86 | 437.33 | 214.22 | 0.43 | 0.11 |
| 10 | 44 | 2.2 | 0.91 | 360.41 | 219.3 | 0.39 | 0.12 |
| 11 | 22 | 2.26 | 0.71 | 344.32 | 206.76 | 0.4 | 0.1 |
| 12 | 44 | 2.2 | 0.75 | 324.14 | 179.85 | 0.4 | 0.1 |
| 13 | 49 | 2.43 | 0.86 | 382.35 | 214.52 | 0.42 | 0.13 |
| 14 | 34 | 2.54 | 0.82 | 426.53 | 214.05 | 0.43 | 0.1 |
| 15 | 22 | 2.21 | 0.83 | 361.32 | 161.11 | 0.43 | 0.1 |
| 16 | 29 | 2.33 | 0.85 | 364.83 | 246.46 | 0.41 | 0.11 |
| 17 | 30 | 2.5 | 0.67 | 380.07 | 144.71 | 0.43 | 0.09 |
| 18 | 28 | 2.22 | 0.77 | 337.29 | 207.78 | 0.39 | 0.11 |
| 19 | 32 | 2.39 | 0.67 | 406.69 | 199.66 | 0.4 | 0.09 |
| 20 | 26 | 2.49 | 0.81 | 443.81 | 240.15 | 0.42 | 0.1 |
| 21 | 17 | 2.01 | 0.76 | 303.06 | 179.65 | 0.35 | 0.11 |
| 22 | 1 | 3 | 0.77 | 266 | 180.2 | 0.54 | 0.12 |
| 23 | 5 | 2.06 | 0.61 | 303.6 | 116.26 | 0.36 | 0.09 |

| Supplementary Table 3. Associations between feeding type and (a) gut microbiome diversity and (b) daily rhythm variables. Results are derived from linear mixed-effects models using breast-fed infants as the reference group. All models were adjusted for infant age and included random intercepts for participant ID. β coefficients represent estimated differences compared to breast feeding infants. | | | |
| --- | --- | --- | --- |
| Outcome Measure | Formula-feed β (p-value) | Mixed-feed β (p-value) | Other feeding β (p-value) |
| (a) Feeding type and gut microbiome diversity | | | |
| Richness | +70.7 (0.0005) | +32.3 (0.021) | +26.7 (0.464) |
| Diversity | +0.33 (0.0004) | +0.18 (0.006) | +0.25 (0.141) |
| Evenness | +0.039 (0.006) | +0.027 (0.006) | +0.037 (0.150) |
| (b) Feeding patterns and daily rhythms | | | |
| Sleep pressure | +0.42 (0.015) | +0.14 (0.350) | +0.33 (0.339) |
| Time since last meal | +0.15 (0.401) | +0.09 (0.577) | +0.55 (0.162) |
| Time since last stool | -0.79 (0.557) | +2.09 (0.080) | -0.69 (0.810) |
| Stool timing | -0.22 (0.687) | +0.41 (0.379) | -0.47 (0.686) |

| 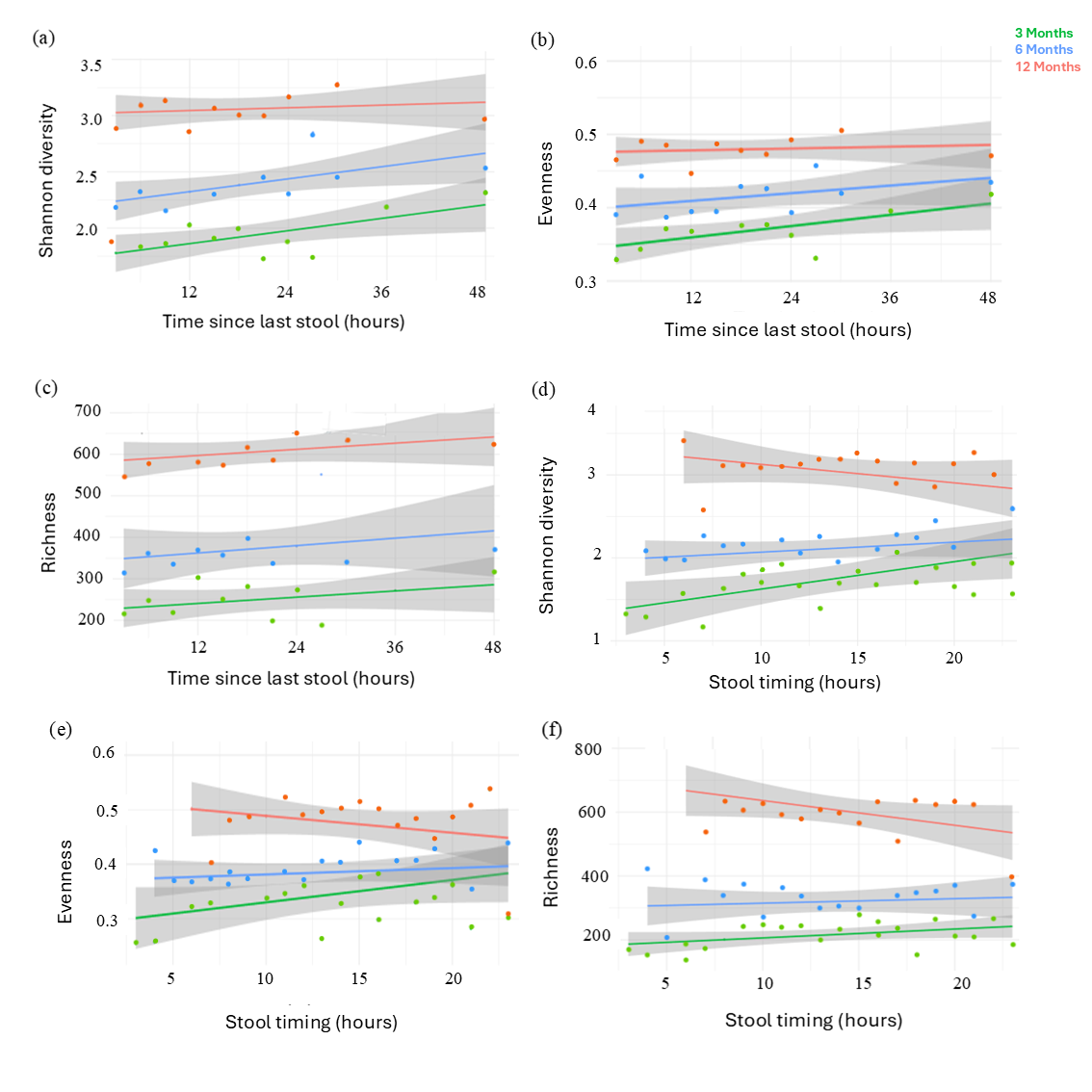 |
| --- |
| Supplementary Figure 1: Stool dynamics and gut microbiome parameters across three timepoints (3, 6, and 12 months). The infant samples are grouped into three-hours intervals in time since the last stool with (a) diversity, (b) evenness, and (c) richness, and one-hour intervals in stool timing variable with (d) diversity, (e) evenness, and (f) richness. |

| 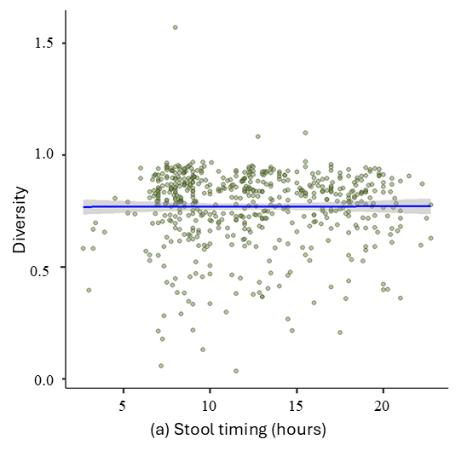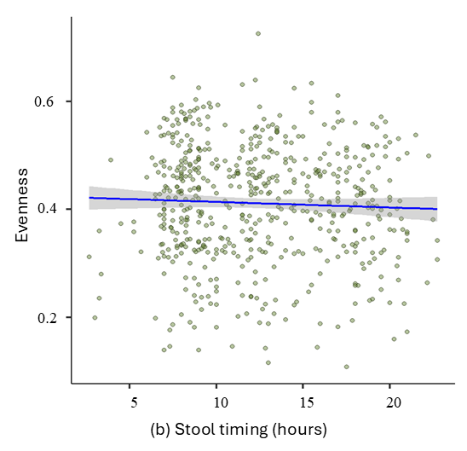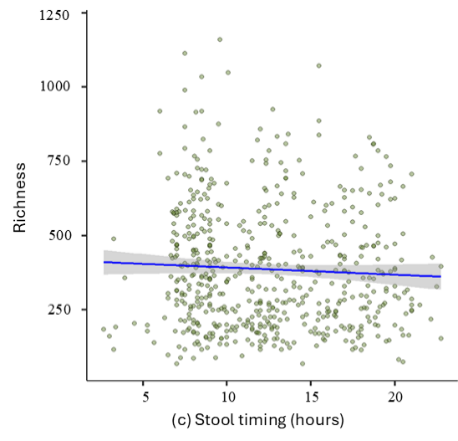 |
| --- |
| Supplementary Figure 2: Mixed-effects model analysis of (a) Diversity, (b) Evenness, and (c) Richness in relation to stool timing in infants. No significant associations were found with diversity and evenness, except a weak negative association with richness (coef = -0.041, p = 0.09). |

| (a)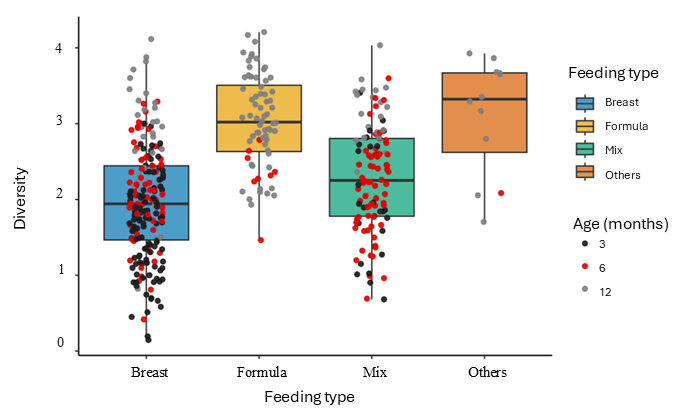 |
| --- |
| (b)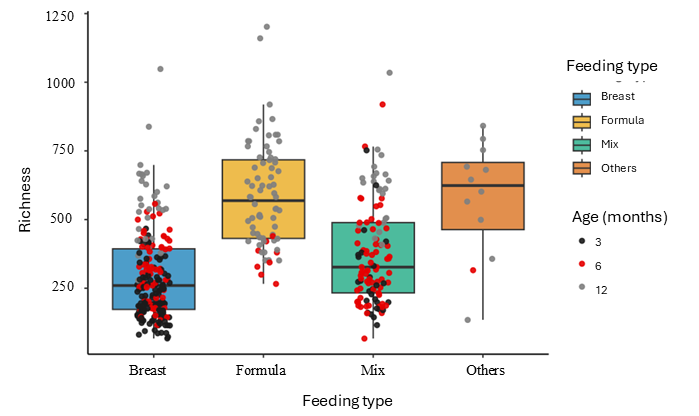 |
| (c)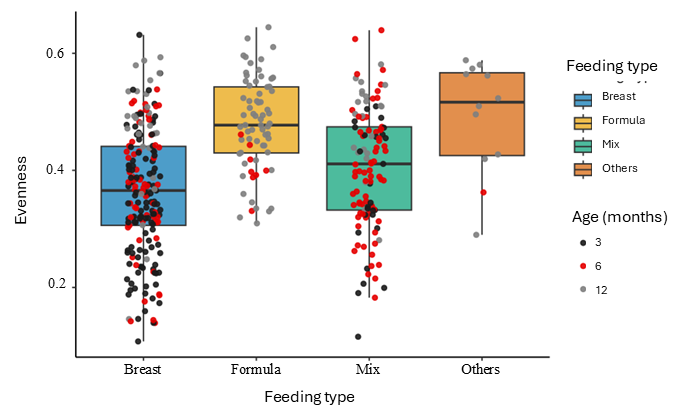 |
| Supplementary Figure 3. Gut microbiome diversity by feeding type. (a) Shannon diversity, (b) richness, and (c) evenness across feeding types (breast, formula, mixed, others). Points represent individual samples coloured by age (black = 3 mo, red = 6 mo, grey = 12 mo). Boxplots show median and interquartile range. Formula-fed infants show higher diversity and richness, with mixed feeding intermediate. |
